# Under-five mortality among mothers employed in agriculture: findings from a nationally representative sample

**DOI:** 10.7717/peerj.710

**Published:** 2015-01-08

**Authors:** Rajvir Singh, Vrijesh Tripathi

**Affiliations:** 1 Cardiology Research Centre, Heart Hospital, Hamad Medical Corporation (HMC), Doha, Qatar; 2 The Faculty of Science and Technology, The University of the West Indies, St. Augustine Campus, Trinidad and Tobago, West Indies

**Keywords:** Mother’s employment, Breastfeeding, NFHS-3, U5MR, Under-five mortality, Agriculture, India

## Abstract

**Background.** India accounts for 24% to all under-five mortality in the world. Residence in rural area, poverty and low levels of mother’s education are known confounders of under-five mortality. Since two-thirds of India’s population lives in rural areas, mothers employed in agriculture present a particularly vulnerable population in the Indian context and it is imperative that concerns of this sizeable population are addressed in order to achieve MDG4 targets of reducing U5MR to fewer than 41 per 1,000 by 2015. This study was conducted to examine factors associated with under-five mortality among mothers employed in agriculture.

**Methods.** Data was retrieved from National Family Household Survey-3 in India (2008). The study population is comprised of a national representative sample of single children aged 0 to 59 months and born to mothers aged 15 to 49 years employed in agriculture from all 29 states of India. Univariate and Multivariate Cox PH regression analysis was used to analyse the Hazard Rates of mortality. The predictive power of child mortality among mothers employed in agriculture was assessed by calculating the area under the receiver operating characteristic (ROC) curve.

**Results.** An increase in mothers’ ages corresponds with a decrease in child mortality. Breastfeeding reduces child mortality by 70% (HR 0.30, 0.25–0.35, *p* = 0.001). Standard of Living reduces child mortality by 32% with high standard of living (HR 0.68, 0.52–0.89, 0.001) in comparison to low standard of living. Prenatal care (HR 0.40, 0.34–0.48, *p* = 0.001) and breastfeeding health nutrition education (HR 0.45, 0.31–0.66, *p* = 0.001) are associated significant factors for child mortality. Birth Order five is a risk factor for mortality (HR 1.49, 1.05–2.10, *p* = 0.04) in comparison to Birth Order one among women engaged in agriculture while the household size (6–10 members and ≥ 11 members) is significant in reducing child mortality in comparison to ≤5 members in the house. Under-five mortality among mothers employed in agriculture in India discriminated well between death and survival (Area Under ROC was 0.75, 95% CI [0.73–0.77]) indicating that the model is good for appropriate prediction of child mortality.

**Conclusion.** In a nationally representative sample of households in India, mother’s age, breastfeeding, standard of living, prenatal care and breastfeeding health nutrition education are associated with reduction in child mortality.

## Background

India is one of the five nations that has the highest number of under-five mortality (United Nations Children’s Fund, 2012). It is important that new measures are taken to drastically bring down under-five mortality from its present 61 per 1,000 to 41 per 1,000 by 2015. The National Family Health Survey (NFHS-3) reports that two-thirds of Indians live in rural areas, have relatively low wealth index, and girls have low levels of education in the rural areas. All these contribute to high child mortality (International Institute for Population Sciences (IIPS) and Macro International, 2007). However, there is no general consensus on whether mothers working in agriculture have any adverse effect on child survival in India. In fact, there have been very few studies that link the employment of mother to under-five mortality.

In their study on the impact of employment upon child mortality in India, Headey, Chiu & Kadiyala (2012) note that there has been a decline in agricultural employment from about 65% in 1991 to about 55% in 2006. They also remark that 83% of working women in rural areas are employed in the agriculture sector. They found that agricultural workers have the lowest body mass index (BMI) compared with non-agricultural workers, even after controlling for wealth, health, education, and location. However, they also note that BMIs are inadequate indicators of maternal health or nutritional status. Their study does not exclusively focus upon women or rural areas or how mother’s employment status in these rural areas affects child mortality.

Analysing maternal health and child mortality in rural India, Pandey (2009) does not find that mother’s employment status affects child survival. However, he notes that mother’s height, weight and anemic status are significant in improving child survival. However, Kishor & Parasuraman (1998) find that a mother’s employment status does affect child survival. They report that the chances of child death at ages 12–47 months are significantly higher when the mothers are employed. The chances of dying at ages 0–11 months are higher only if the mother is employed in or outside the home for wages. They conclude that during childhood, the odds of dying increase for male and female children if the mother works. For boys, the increase is greatest if the mother works outside the home for wages, and for girls, if the mother works at home. However, Kishor & Parasuraman (1998) do not focus on mothers employed in agriculture.

Other studies also identify a higher risk of mortality in male children among mothers working away from home, especially in agriculture (Basu & Basu, 1991; Gunasekaran, 2008). A 1983 World Health Organisation (WHO) study from Guatemala reveals that commercialization of agriculture worsened child mortality in affected areas relative to the rest of the country (Hugo, 1983). Another study from Kenya reports that child mortality is higher for mothers working in agriculture compared to those mothers not working. They attribute this to the nature of employment and the fact that women are not available to care for the children for long periods of time (Mustafa & Odimegwu, 2008).

Hence, it is projected that mothers engaged in agricultural work in the fields are at a disadvantage in ensuring child survival due to socio-demographics, nutritional deficiencies in diets and limited access to health facilities in the rural areas. India is an agricultural economy, with a large proportion of India’s population based in rural areas. Women employed in the agricultural sector are contributors to this economy and it is imperative that they receive the benefits of growth in the Indian economy. The study reviews determinants of under-five mortality among mothers engaged in agriculture with a view to reduce child mortality in India.

## Materials and Methods

### Data

Data for the study was downloaded from the Demographic and Health Survey (DHS) website. The nationally representative household survey, conducted by the International Institute for Population Sciences and Macro International from November 2005 to August 2006, is based upon stratified, multistage cluster sampling with two stages at rural and three stages at urban level. All women between the ages of 15–49 interviewed in 29 states in 5 regions were asked to self-report on the birth and death history. Data is extracted for all child births that had taken place in the 59 months preceding the survey, and time in months is noted for each reported death during this period. These procedures generate a sample of 124,385 female participants at 95% response rate with data on 56,438 births. Those alive at the date of survey are censored for the analysis, and the duration of live birth in months is calculated from the date of birth to the date of survey of that subject for each birth order separately. Data is further extracted to get a sub-sample for our study that consists of mothers employed in the agricultural sector. Information on first to fifth parity is available for a total of 12,291 births that took place in the 59 months preceding the survey. Parity six and above are too few and hence excluded from the study.

### Measures

Family characteristics are assessed at each index child using single items regarding region, wealth index, standard of living, religion, caste, size of household and father’s education and occupation. National regions of residence are defined as North, Central, East, North East, West and South. These categorizations are based on those created by the International Institute for Population Sciences (IIPS). The IIPS took information on 30 household items and categorised the household into five quintiles with Poorest to the Wealthiest on a scale of 1 to 5. Religion is denoted as Hindu, Muslim, Christian and others. Caste is categorised as scheduled castes, scheduled tribes, other backward castes and others. Size of household is categorised as ≤5 members, 6–10 members and ≥11 members in our study. Parental education is classed into no formal education, primary education and secondary education and above, including graduation and post-graduation. Father’s occupation is categorised as no employment, employment in agriculture and others.

Characteristics of the child are assessed by gender and size at birth. Child size at birth is categorized as very large, larger than average, average, smaller than average and very small. These categories are combined to above average, average and below average. Characteristics of the mothers under study are her age, education, BMI, and anemia. Since the mother’s age at index child shows a curvilinear relation with child mortality, the mother’s age is coded as ≤21 years, 22–23 years, 24–26 years and >26 years to satisfy linearity and proportionality assumptions for Cox PH regression analysis. Body Mass Index (BMI) is based on height and weight measurements obtained by the interviewer at the time of interview and classed as underweight, normal and obese, according to WHO guidelines. Anemia is categorised as severe, moderate and no anemia.

Utilization of health care services are characterised as before and after delivery. All answers related to access to health facilities are coded according to the NFHS guidelines. Place of antenatal care is coded as at home, Government hospital, or private hospital/village/Non-governmental Organisation. Received Prenatal care, Pregnancy Health Education Index, and Breastfeeding Health Nutrition Education are coded as yes/no. Place of delivery is classed as home or hospital. Birth order is taken from Birth Order 1 to 5. Breastfeeding, Immunization and whether they were told of complications in pregnancy are also coded as yes/no.

### Data analysis and statistical methods

Demographic characteristics and outcome measures are described using weighted percentages. Univariate Cox Proportional Hazard (PH) analysis is used to determine factors associated with child mortality. Kaplan–Meier curves are used to satisfy PH assumption. Significant variables at univariate analysis with *p*-value ≤ 0.10 and modified appropriate categories are considered for multivariate Cox PH regression after checking co-linearity among the explanatory variables. Forward stepwise Cox PH method is applied to assess significant explanatory variables of child mortality among mothers employed in agriculture and adjusted Hazard Ratios (HR) with 95% confidence intervals (CI) are presented. Cross-validation of confidence intervals calculated for HRs derived by multivariate Cox Proportional Hazard analysis are internally validated via bootstrapping re-sampling methods using 100 re-samples (Cox, 1972; Harrell, 2001). The predictive accuracy of the model is tested through calculating the area under the receiver operating characteristic (AUROC) curve (Tripepi et al., 2009). The ROC curve is used to quantify the overall accuracy of a model in differentiating outcome groups and is a good measure of its predictive ability. The area under the curve ranges from 0.5 to a theoretical maximum of 1. Acceptable discrimination is represented by an area under the curve of 0.70–0.79, and good discrimination by an area under the ROC curve >0.80. Perfect discrimination corresponds to a c-statistic of 1 and is achieved if the scores for all the cases are higher than those for all the non-cases, with no overlap. The nonparametric method does not make any assumptions about the distributions of test results between the two groups. It is used to calculate Standard Error (SE) and 95% CI of the ROC. *P*-value ≤ 0.05 (two tailed) is considered for statistical significance. SPSS 20.0 statistical package is used for the analysis (IBM Corp., 2012).

No identifying information is available in the data precluding the need for any ethical approvals. We wish to thank the DHS for making this data available for our study.

## Results

### Population characteristics

Family characteristics of the population are described in Table 1. Data is available for a total of 9,532 births in un-weighted and 12,291 births in a weighted sample of mothers employed in the agricultural sector. Of all births, 31.2% occur in the Central region, 23.6% in the East region, 14.9% in the West region, 14.4% in the South region, 14.2% in the North region, and 1.7% in the North East region. 70% belong to poor and poorest wealth index while only 9.6% are in richer and richest wealth index. Nearly 43.6% households belong to low and 40% to medium standard of living. 90% are Hindu, 5.9% are Muslim, 1.8% are Christian and 2.2% are classed as others. 23.1% belong to scheduled castes, 19.1% belong to scheduled tribes and 46.3% belong to other backward castes. 54.3% , 37% and 8% deaths occur in households with ≤5 members, 6–10 members and ≥11 members, respectively. 55% of fathers received education either less than or up to primary school. 57% of fathers are employed in agriculture while the rest are in service elsewhere.

**Table 1:** Characteristics of family background according to child status.

Name of covariates	Category	Alive	Dead	Total
		11,379	912	12,291
Region	North^a^	1,613(14.2)	129(14.1)	1,742(14.2)
	Central^b^	3,519(30.9)	321(35.2)	3,840(31.2)
	East^c^	2,687(23.6)	215(23.5)	2,902(23.6)
	North East^d^	191(1.7)	15(1.6)	206(1.7)
	West^e^	1,705(15.0)	126(13.8)	1,831(14.9)
	South^f^	1,664(14.6)	107(11.7)	1,771(14.4)
Wealth index	Poorer & Poorest	7,943(69.8)	692(75.9)	8,635(70.3)
	Middle	2,305(20.3)	168(18.4)	2,473(20.1)
	Richer & Richest	1,131(9.9)	52(5.7)	1,183(9.6)
Standard of living	Low	4,459(43)	417(50.7)	4,876(43.6)
	Medium	4,171(40.2)	321(39)	4,492(40.1)
	High	1,736(16.7)	85(10.3)	1,821(16.3)
Mother’s religion	Hindu	10,257(90.2)	803(88.1)	11,060(90.1)
	Muslim	664(5.8)	64(7.0)	728(5.9)
	Christian	191(1.7)	20(2.2)	217(1.8)
	Others	250(2.2)	24(2.6)	274(2.2)
Caste	Scheduled caste	2,569(22.8)	239(26.7)	2,808(23.1)
	Scheduled tribe	2,140(19)	184(20.6)	2,324(19.1)
	Other backward castes	5,257(46.7)	367(41.1)	5,624(46.3)
	Others	1,280(11.4)	104(11.6)	1,384(11.4)
Size of household	≤5 Members	4,200(36.9)	495(54.3)	4,695(38.2)
	6–10 Members	5,763(50.6)	337(37)	6,100(49.6)
	≥11 Members	1,416(12.4)	80(8.8)	1,496(12.2)
Father’s education	No education	4,329(38.5)	363(40.5)	4,692(38.7)
	Primary	1,888(16.8)	175(19.5)	2,063(17.0)
	Secondary & above	5,024(41.6)	359(40.0)	5,383(44.3)
Father’s occupation	No work	73(0.6)	12(1.3)	85(0.7)
	Service	4,751(41.9)	382(41.9)	5,133(41.9)
	Agriculture	6,515(57.5)	518(56.8)	7,033(57.4)

**Notes.**

Figures in parentheses are percentages of the total study population.

a New Delhi, Haryana, Himachal Pradesh, Jammu/Kashmir, Punjab, Rajasthan, Uttaranchal.

b Madhya Pradesh, Uttar Pradesh, Chattisgarh.

c Bihar, Orissa, West Bengal, Jharkhand.

d Arunachal Pradesh, Assam, Manipur, Meghalaya, Mizoram, Nagaland, Sikkim, Tripura.

e Goa, Gujarat, Maharashtra.

f Andhra Pradesh, Karnataka, Kerala, Tamil Nadu.

Table 2 presents the characteristics of the child and mother. Of the 12,291 total births 457 male and 455 female children died during the chosen period of study. 22% of children are born below average size. Nearly 68% of mothers have no formal education while 15% only attain primary education. 45.5% of mothers are underweight while only 3.2% are obese. Nearly 36% of mothers are not anemic, 41.6% are mildly anemic while 20.4% are moderately anemic and 1.9% are severely anemic.

**Table 2:** Characteristics of mother and child according to child status.

Name of covariates	Category	Alive	Dead	Total
		11,379	912	12,291
Child’s gender	Male	5,965(52.4)	457(50.1)	6,422(52.2)
	Female	5,414(47.6)	455(49.9)	5,869(47.8)
Child birth size	Above average	2,494(22.2)	192(22.1)	2,686(22.2)
	Average	6,322(56.4)	387(44.5)	6,709(55.5)
	Below average	2,401(21.4)	291(33.4)	2,692(22.3)
Mother’s age	≤ 21 Yrs	2,261(19.9)	224(24.1)	2,485(20.2)
	22–23 Yrs	1,946(17.1)	162(17.7)	2,108(17.1)
	24–26 Yrs	2,949(25.9)	242(26.5)	3,191(26.0)
	>26 Yrs	4,224(37.1)	285(31.2)	4,509(36.7)
Mother’s education	No education	7,681(67.5)	682(74.8)	8,363(68)
	Primary	1,734(15.2)	151(16.6)	1,885(15.3)
	Secondary & above	1,964(17.3)	80(8.8)	2,044(16.6)
Mother’s BMI	Underweight^a^	5,045(45.6)	385(43.6)	5,430(45.5)
	Normal^b^	5,651(51.1)	479(54.2)	6,130(51.3)
	Obese^c^	365(3.3)	19(2.2)	384(3.2)
Anemia level of mother	Severe	202(1.9)	23(2.6)	225(1.9)
	Moderate	2,165(19.9)	242(27.7)	2,407(20.4)
	Mild	4,589(41.1)	316(36.2)	4,905(41.6)
	Not anemic	3,949(36.2)	292(33.4)	4,241(36.0)

**Notes.**

a <18.5 kg/m^2^.

b 18.5–24.9 kg/m^2^.

c ≥ 25 kg/m^2^.

Utilization of the health care services are presented in Table 3. For antenatal care, the majority (56%) go to government hospitals, 31% go to private hospitals while 13% receive care at home. 48.7% receive prenatal care; 48.6% receive pregnancy health nutrition education; and 10.5% receive breastfeeding health nutrition education. Over 77% are home deliveries while 23% are delivered in hospitals. 99% receive assistance at delivery; pregnancy complications occur in 8.7%. Children receive breastfeeding in 70% of cases. Of the total births, 24.6% are of Birth Order 1, 27.4% are Birth Order 2, 21.3% are Birth Order 3, 15.4% are Birth Order 4 and 11.2% are Birth Order 5. Immunization services are used by 7.1%.

**Table 3:** Utilization of health services according to child status.

Name of covariates	Category	Alive	Dead	Total
		11,379	912	12,291
**Before delivery characteristics**				
Place of antenatal care	At home	735(912.9)	43(15.4)	778(13.0)
	Govt. hosp.	3,196(56.1)	148(52.9)	3,344(56.0)
	Private hosp./village/NGO	1,762(31.0)	90(32.0)	1,852(31.0)
Prenatal care	No	5,678(49.9)	631(69.1)	6,309(51.3)
	Yes	5,701(50.1)	282(30.9)	5,983(48.7)
Pregnancy health nutrition education	No	1,740(51.1)	116(56.0)	1,856(51.4)
	Yes	1,666(48.9)	91(44.0)	1,757(48.6)
Breastfeeding health nutrition education	No	10,127(89.0)	871(95.5)	10,998(89.5)
	Yes	1,253(11.0)	41(4.5)	1,294(10.5)
**Delivery and post-delivery related characteristics**				
Place of delivery	At hospital	2,615(23.0)	195(21.6)	2,810(22.9)
	At home	8,764(77.0)	707(78.4)	9,471(77.1)
Assistance at delivery	No	82(0.7)	4(0.4)	86(0.7)
	Yes	11,294(99.3)	898(99.6)	12,192(99.3)
Pregnancy complication	No	10,364(91.1)	856(93.9)	11,220(91.3)
	Yes	1,015(8.9)	56(6.1)	1,071(8.7)
Breastfeeding	No	3,079(27.1)	529(58.0)	3,608(29.4)
	Yes	8,300(72.9)	383(42.0)	8,683(70.6)
Birth order	1st	2,747(24.1)	281(30.8)	3,028(24.6)
	2nd	3,118(27.4)	249(27.3)	3,367(27.4)
	3rd	2,476(21.8)	145(15.9)	2,621(21.3)
	4th	1,756(15.4)	142(15.6)	1,898(15.4)
	5th	1,283(11.3)	95(10.4)	1,378(11.2)
Ever immunization	No	10,561(92.8)	854(93.6)	11,415(92.9)
	Yes	818(7.2)	58(6.4)	876(7.1)

Predominantly, mothers working in agriculture are Hindu, belong to other backward castes, receive less than or up to primary education, and are in poor or poorest households with 6–10 members. The majority of the children born are average or above average size. Most mothers receive no prenatal care and are not told of pregnancy complications. Most of the children are born at home and never immunized. A large number of children who die belong to households with less than or equal to 5 members and were not breastfed.

### Univariate analysis

Univariate Analysis of family characteristics associated with child mortality are represented in Table 4. Households at the poorer and poorest wealth indices are risk factors compared with middle (HR 0.72, 0.60–0.88, 0.001) and richer and richest (HR 0.49, 0.37–0.66, *p* = 0.001) wealth indices. Medium Standard of Living (HR 0.75, 0.63–0.89, *p* = 0.001) and high standard of living (HR 0.54, 0.42–0.70, *p* = 0.001) act as protective factors with rise in standard of living corresponding to reduction in child mortality. Other backward castes (HR 0.80, 0.64–0.98, *p* = 0.03) are at less risk compared to scheduled castes. There is no statistically significant difference between scheduled castes and scheduled tribes. Size of household significantly reduces child mortality for households with 6–10 members (HR 0.57, 0.49–0.67, *p* = 0.001) and for households with ≥11 members (HR 0.57, 0.44–0.77, *p* = 0.001) in comparison to ≤5 members. Region, religion, the father’s education and occupation are not significant factors.

**Table 4:** Univariate Cox PH regression analysis for characteristics of family background.

Name of covariates	Category	Univariate HR (95% CI)
Region	North	1
	Central	1.25(1.00–1.57)
	East	1.09(0.84–1.41)
	North-East	0.78(0.60–1.02)
	West	0.99(0.73–1.34)
	South	0.77(0.57–1.06)
Wealth index	Poorer & poorest	1
	Middle	0.72(0.60–0.88)^**^
	Richer & richest	0.49(0.37–0.66)^**^
Standard of living	Low	1
	Medium	0.75(0.63–0.89)^**^
	High	0.54(0.42–0.70)^**^
Mother’s religion	Hindu	1
	Muslim	1.17(0.88–1.55)
	Christian	0.82(0.64–1.06)
	Others	0.88(0.60–1.30)
Caste	Scheduled caste	1
	Scheduled tribe	0.82(0.66–1.02)
	Other backward castes	0.80(0.64–0.98)^*^
	Others	0.78(0.60–1.03)
Size of household	≤5 Members	1
	6–10 Members	0.57(0.49–0.67)^*^
	>=11 Members	0.58(0.44–0.77)^*^
Father’s education	No education	1
	Primary	1.07(0.86–1.33)
	Secondary & above	0.87(0.73–1.03)
Father’s occupation	No work	1
	Service	0.79(0.38–1.68)
	Agriculture	0.82(0.39–1.72)

**Notes.**

* *p* ≤ 0.05.

** *p* < 0.001.

Table 5 presents the Univariate analysis of characteristics of child and mother associated with child mortality. Male and female children are equally at risk. Below average child size is a risk factor (HR 1.65, 1.32–20.06, *p* = 0.001) for child mortality. Risk of child mortality decreases with increase in the mother’s age from less than 21 years to above 26 years. The mother’s education is a protective factor with secondary level and higher acting as a significant protective factor (HR 0.49, 0.38–0.61, *p* = 0.001). With improvements in the mother’s anemic level, deaths are fewer in number. Not having anemia is a protective factor (HR 0.60, 0.35–1.0, *p* = 0.05).

**Table 5:** Univariate Cox PH regression analysis for mother and child characteristics.

Name of covariates	Category	Univariate HR (95% CI)
Child’s gender	Male	1
	Female	1.05(0.90–1.22)
Child birth size	Above average	1
	Average	0.90(0.72–1.10)
	Below average	1.65(1.32–2.00)^**^
Mother’s age	≤21 Yrs	1
	22–23 Yrs	0.80(0.63–1.02)^*^
	24–26Yrs	0.72(0.58–0.90)^**^
	>26 Yrs	0.56(0.45–0.68)^**^
Mother’s education	Father’s education	1
	Primary	0.90(0.73–1.09)
	Secondary & above	0.49(0.38–0.61)^**^
Mother’s BMI	Underweight	1
	Normal	1.06(0.90–1.25)
	Obese	0.72(0.44–1.18)
Anemic level of mother	Severe	1
	Moderate	0.94(0.56–1.60)
	Mild	0.66(0.39–1.11)
	Not anemic	0.60(0.35–1.00)^*^

**Notes.**

* *p* ≤ 0.05.

** *p* < 0.001.

Univariate analysis of utilization of health care services are described in Table 6. Prenatal care decreases mortality by 53% (HR 0.47, 0.39–0.55, *p* = 0.001). Breastfeeding health nutrition education reduces mortality by 59% (HR 0.41, 0.28–0.59, *p* = 0.001). Being told of pregnancy complications decreases child mortality (HR 0.59, 0.42–0.81, *p* = 0.001). Breastfeeding is a protective factor, reducing the risk of child mortality by 64% (HR 0.36, 0.30–0.41, *p* = 0.001). Birth order is a statistically significant factor with survival at Birth Order 1 being at significant risk compared to higher birth orders. Mortality significantly decreases with increasing birth order. Place of antenatal care services, BMI, pregnancy health nutrition education, place of delivery, ever immunization and assistance at delivery did not present themselves as significant factors for under-five mortality among mothers employed in agriculture.

**Table 6:** Univariate Cox PH regression analysis of utilization of health services.

Name of covariates	Category	Univariate HR (95% CI)
**Before delivery characteristics**		
Place of antenatal care	At Home	1
	Govt. hospital	0.91(0.60–1.40)
	Private hospital/village/NGO	0.85(0.54–1.36)
Prenatal care	No	1
	Yes	0.47(0.39–0.55)^**^
Pregnancy health nutrition education	No	1
	Yes	0.90(0.66–1.24)
Breastfeeding health nutrition education	No	1
	Yes	0.41(0.28–0.59)^**^
**Delivery and post-delivery related characteristics**		
Place of delivery	At home	1
	At hospital	0.96(0.80–1.15)
Assistance at delivery	No	1
	Yes	1.67(0.63–4.47)
Pregnancy complication	No	1
	Yes	0.59(0.42–0.81)^**^
Breastfeeding	No	1
	Yes	0.36(0.30–0.41)^**^
Birth order	1st	1
	2nd	0.73(0.60–0.90)^**^
	3rd	0.61(0.48–0.76)^**^
	4th	0.76(0.60–0.96)^*^
	5th	0.77(0.59–1.00)
Ever immunization	No	1
	Yes	0.97(0.71–1.33)

**Notes.**

* *p* ≤ 0.05.

** *p* < 0.001.

### Multivariate analysis

The results of multivariate analysis of factors associated with child mortality are shown in Table 7. A high standard of living significantly reduces child mortality (HR 0.68, 0.52–0.89, *p* = 0.001) in comparison to a low sandard of living. Size of household reduces mortality in households with 6–10 members by 40% (HR 0.60, 0.50–0.72) and in households ≥11 by 34% (HR 0.66, 0.48–0.91), respectively, in comparison to households with ≤5 members. Increase in the mother’s age corresponds with reduction in child mortality. Receiving prenatal care (HR 0.40, 0.34–0.48, *p* = 0.001) is a highly significant protective factor in child mortality. Breastfeeding health nutrition education is a significant protective factor (HR 0.45, 0.31–0.66, *p* = 0.001). Breastfeeding reduces child mortality significantly by 70% (HR 0.30, 0.25–0.35, *p* = 0.001). Birth order is not a statistically significant factor in multivariate analysis though Birth Order 5 is at a slightly higher risk than Birth Order 1.

**Table 7:** Multivariate Cox PH regression analysis for covariates associated with child mortality.

Name of covariates	Category	Multivariate adjusted HR (95% CI)
Standard of living	Low	1
	Medium	0.86(0.72– 1.02)
	High	0.68(0.52 –0.89)^**^
Size of household	≤5 members	1
	6–10 members	0.60(0.50–0.72)^*^
	> = 11 members	0.66(0.48–0.91)^*^
Mother’s age	≤21 Yrs	1
	22–23 Yrs	0.77(0.60–1.00)
	24–26 Yrs	0.61(0.48 –0.79)^**^
	>26 Yrs	0.39(0.30–0.51)^**^
Prenatal care	No	1
	Yes	0.40(0.34–0.48)^**^
Breastfeeding health nutrition education	No	1
	Yes	0.45(0.31–0.66)^**^
Breastfeeding	No	1
	Yes	0.30(0.25–0.35)^**^
Birth order	1st	1
	2nd	0.87(0.70–1.09)
	3rd	0.82(0.62–1.07)
	4th	1.30(0.97–1.76)
	5th	1.49(1.05–2.10)^*^

**Notes.**

* *p* < 0.05.

** *p* < 0.01.

**Figure 1:**
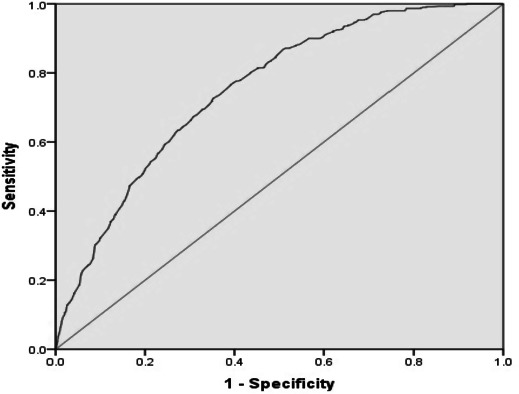
Receiver operating characteristic (ROC) curve for predicting child mortality among mothers working in agriculture. Area Under the Curve (ROC) = 0.75, 95% CI (0.73–0.77).

The area under the receiver operating characteristic (ROC) curve, measuring the ability of the model to discriminate mortality outcomes (death/no death) and to evaluate the model’s practical ability for correctly discriminating a subject’s outcome is shown in Fig. 1. Bootstrapping analysis shows only a little bias in estimated parameters. The Area Under ROC is 0.75 (95% CI [0.73–0.77]), indicating that the developed model is fair enough for appropriate prediction of child mortality containing true area between 0.73 and 0.77 with 95% surety.

## Discussion

Among the family characteristics, wealth index, standard of living, other backward caste and size of household emerge as significant in univariate analysis. Our study finds that the mother’s standard of living has substantial impact on child mortality in univariate and multivariate analysis. This is supported by a study in Bangladesh (Uddin, Hossain & Ullah, 2009). Low standard of living was a risk factor as compared with high standard of living. This is in keeping with the general notion that poverty affects child survival adversely. Other backward castes dominate the agricultural sector and are statistically significant in univariate analysis. However, caste is not a statistically significant factor in multivariate analysis. Size of household emerges as a significant factor in multivariate analysis. This is consistent with existing literature that suggests larger households show less under-five mortality (Headey, Chiu & Kadiyala, 2012). This can be related to the fact that often a large household has elders who take care of the young child while the mother goes to work.

A study from Bangladesh reports that the child mortality rate is highest for children whose fathers’ occupation is agriculture (Uddin, Hossain & Ullah, 2009). A study in Pakistan reveals that the children of fathers in salaried employment have lower risks of child mortality than those engaged in agriculture or are unemployed (Kiani, 1992). However, the father’s education or employment did not reveal itself as a significant factor in univariate or multivariate analysis in our study.

Among the characteristics of the child, the child’s gender appears to be statistically insignificant. The NFHS-3 report states that after the first month of life and before five years of age, girls face a higher mortality risk than boys (International Institute for Population Sciences (IIPS) and Macro International, 2007). However, there is no gender bias in the survival of the girl child among mothers engaged in agriculture. Family support mechanisms, available in large households with more than 6 members, lessen gender biases in the survival of female children (Murthi, Guio & Dreze, 1995). Below average birth size is a significant factor in univariate analysis only.

In our study of the characteristics of the mother, education to secondary and above levels was a significant factor in univariate analysis only. Tulasidhar analysed Indian census data to conjecture that there was a link between primary education up to seven years, child mortality and female labour force participation, but the link between more than seven years of education and child mortality was not strong. He recommended that extending primary education would reduce child mortality (Tulasidhar, 1993; Kiros & Hogan, 2001). Our study points that mere literacy or even education up to the primary level cannot help reduce child mortality. Women with little or no education, in fact, may require more counselling and information during pregnancy.

Among the studied variables of health care, multivariate analysis reveals the absence of prenatal care as a significant factor in under-five mortality. Prenatal care is not utilized in rural areas to the extent that it is utilized in the urban areas. The place of antenatal care is not a significant factor in either univariate or multivariate analysis. However, prenatal care is a significant factor in univariate and multivariate analysis, irrespective of whether it is provided at home or in government or private hospitals. Moreover, maternal education also plays an important part in utilization of antenatal care (Kumar et al., 2013; Chalasani, 2012; Mazumdar, 2010; Pradhan & Arokiasamy, 2010; Gakidou et al., 2010; Boone & Zhan, 2006). Hence, it is important that continued efforts be made to educate girls to optimize the awareness and utilization of available health facilities among young mothers working in agriculture.

Breastfeeding health nutrition education reported low information, education and communication (IEC). This factor is significant in univariate and multivariate analysis. Furthermore, the NFHS-3 reports that “Although breastfeeding is nearly universal in India, only 46% of children under 6 months are exclusively breastfed, as WHO recommends. In addition, only 55% are put to the breast within the first day of life…” (International Institute for Population Sciences (IIPS) and Macro International, 2007). Most mothers did not receive any information on the benefits of breastfeeding. This, along with social taboos regarding feeding the baby the yellow milk in the first few days, is not addressed. This factor along with breastfeeding is a significant factor in our univariate and multivariate analysis. Breastfeeding has been recognised as a significant factor in ensuring child survival (Uddin, Hossain & Ullah, 2009). Our findings indicate that though it may appear that breastfeeding is nearly universal in India, our specific population, engaged in agriculture, does not breastfeed due to various reasons, including going to labour in the agricultural fields. Since the mothers are working in the fields, the children are often left behind at home in the care of an elder or an older child. It is recommended that there should be incentives equivalent to those provided as maternity benefits to the working woman in the organised sectors of employment such that the woman may have the choice of staying home to look after the baby. Further, these incentives/ benefits should be continued for at least a year, and the mother may be counselled for immunization in order to reduce under-five mortality.

Though birth order was a significant factor in univariate analysis, it did not emerge as a significant factor in multivariate analysis. Mishra & Retherford (2008) found that three-quarters of births in rural India take place at home. However, place of delivery and assistance at birth did not appear as significant factors in univariate or multivariate analysis.

The developed model demonstrates fair discrimination, indicating that the model fits the data. The AUROC is equivalent to the probability that the measure or predicted risk is higher for mortality than for survival in the study.

## Limitations

Breastfeeding and immunization are taken as single yes/no variables that measure ever/never use. It is surprising that a large population reported in the negative, giving credence that they were never counselled regarding the benefits of either breastfeeding or immunization. Furthermore, the paper does not identify information on morbidity and mortality of mothers and children that could offer more insights.

## Conclusion

The study identifies mothers involved in agriculture as potential candidates for counselling for drastic reduction in child mortality rates in India. It also recommends that since agriculture contributes substantially to India’s Gross Domestic Product (The World Bank), institutionalised benefits such as paid maternity leave be extended to the agricultural sector.
